# Synthesis and Evaluation of Thick Films of Electrochemically Deposited Bi_2_Te_3_ and Sb_2_Te_3_ Thermoelectric Materials

**DOI:** 10.3390/ma10020154

**Published:** 2017-02-10

**Authors:** Nguyen Huu Trung, Kei Sakamoto, Nguyen Van Toan, Takahito Ono

**Affiliations:** 1 Graduate School of Engineering, Tohoku University, Sendai 980-8579, Japan; ono@nme.mech.tohoku.ac.jp; 2 Micro/Nano-Machining Education and Research Center, Tohoku University, Sendai 890-8579, Japan; kei_sakamoto@m.tohoku.ac.jp; 3 Microsystem Integration Center (μSIC), Tohoku University, Sendai 980-8579, Japan; nvtoan@nme.mech.tohoku.ac.jp

**Keywords:** thermoelectric materials, electrochemical deposition, annealing effects, thick films, thermoelectric power generators

## Abstract

This paper presents the results of the synthesis and evaluation of thick thermoelectric films that may be used for such applications as thermoelectric power generators. Two types of electrochemical deposition methods, constant and pulsed deposition with improved techniques for both N-type bismuth telluride (Bi_2_Te_3_) and P-type antimony telluride (Sb_2_Te_3_), are performed and compared. As a result, highly oriented Bi_2_Te_3_ and Sb_2_Te_3_ thick films with a bulk-like structure are successfully synthesized with high Seebeck coefficients and low electrical resistivities. Six hundred-micrometer-thick Bi_2_Te_3_ and 500-µm-thick Sb_2_Te_3_ films are obtained. The Seebeck coefficients for the Bi_2_Te_3_ and Sb_2_Te_3_ films are −150 ± 20 and 170 ± 20 µV/K, respectively. Additionally, the electrical resistivity for the Bi_2_Te_3_ is 15 ± 5 µΩm and is 25 ± 5 µΩm for the Sb_2_Te_3_. The power factors of each thermoelectric material can reach 15 × 10^−4^ W/mK^2^ for Bi_2_Te_3_ and 11.2 × 10^−4^ W/mK^2^ for Sb_2_Te_3_.

## 1. Introduction

Among thermoelectric materials, N-type bismuth telluride (Bi_2_Te_3_) and P-Type antimony telluride (Sb_2_Te_3_) are attractive due to their high performances for thermoelectric power generation applications near room temperature. Thick films of thermoelectric material with a high Seebeck coefficient and low electrical resistivity are highly desired to fabricate high performance micro power generator devices. There are many methods aimed at synthesizing these materials [1,2,3,4]. Electrochemical deposition is one of potential methods for thick film deposition [3]. In this method, it is reported that optimized techniques can enable the synthesis of materials with high quality morphology and compactness. One of them is a pulsed deposition method. The advantage of pulsed deposition is first demonstrated in Reference [5]. According to this reference, thick and compact Bi_2_Te_3_ films are deposited at a rate of 50 µm/h using electrolytes with high concentrations of 80 mM Bi^3+^ and 90 mM Te^2−^. However, the Seebeck coefficients of synthesized films are very low (~−40 µV/K). Another option is an addition of non-aqueous electrolytes that leads to high ion solubilities. Many non-aqueous additives have been researched for Bi_2_Te_3_: ethylene glycol [6,7] dimethyl sulfoxide [8,9], ethanol [10], ionic liquids (choline chloride) [11,12], molten salt (AlCl_3_–NaCl–KCl) [13], and polyvinyl alcohol [14]. Additionally, the appearance of the Bi_2_Te_3_ soluble anode is proven to enhance a homogeneous composition of deposited films in References [14,15,16]. In this work, another possibility of a deposition of thick and stable thermoelectric films has been demonstrated. The electrolytes are used with a low concentration of cations and ions, resulting in a controlled low deposition rate. The concentrations of Bi^3+^ and Te^2−^ are 4 mM and 3.6 mM, respectively. Therefore, the amorphous material is easily crystallized during the pulsed deposition. By this method, without the necessity of using non-aqueous additives and a soluble anode, the Seebeck coefficients of synthesized Bi_2_Te_3_ thick films are more improved than that of using high concentration electrolytes [5], and the same as using a soluble anode (~−80 µV/K) [15,16]. Additionally, this mechanism is successfully applied for not only N-type Bi_2_Te_3_ but also P-type Sb_2_Te_3_ thick films.

## 2. Experimental

### 2.1. Sample Preparation

**Figure 1:**
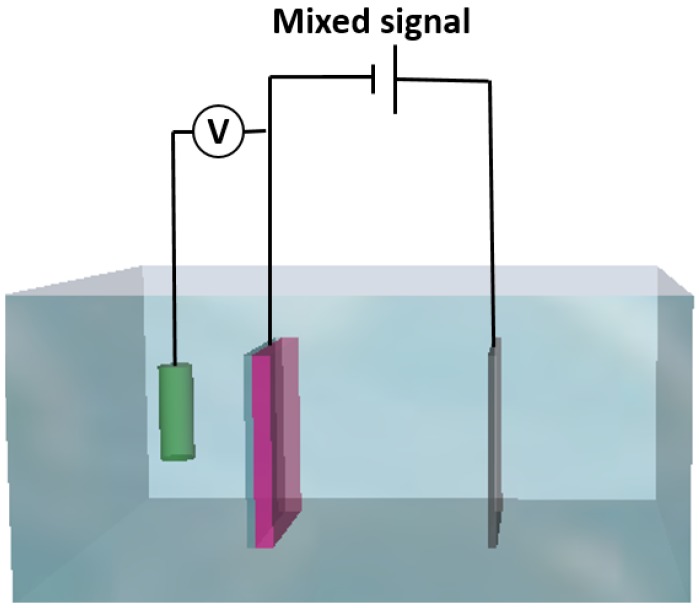
Electrochemical deposition system.

The sample preparation process begins from a 300-µm-thick silicon substrate. Cr-Au films with thicknesses of 10 nm and 150 nm, respectively, are deposited on a silicon substrate by sputtering. A three-electrode electrochemical deposition method is used as follows: the 300-µm-thick silicon substrate with a Cr/Au seed layer is used as the working electrode, a platinum mesh is used as a counter electrode, and Ag/AgCl with a 3 M KCl (Potassium Chloride) solution is used as a reference electrode. A schematic of deposition system is shown in Figure 1.

### 2.2. Electrochemistry

Electrochemical deposition of the BiTe is performed and compared using constant and pulsed waveform methods at room temperature with slow stirring (60 rpm). According to previous discussion, the usage of electrolytes with high concentrations of ions and cations has the advantage in high deposition rate. However, the deposition encounters the obstacle to grow high quality crystal films. Therefore, a low concentration electrolyte has been used to mitigate this negative behavior. The electrolyte solution consists of 4 mM Bi^3+^, 3.6 mM HTeO^2+^, and 1 M HNO_3_. The solution is prepared from the following steps. At first, both Bi_2_O_3_ and TeO_2_, are dissolved in nitric acid. Deionized (DI) water is added to both solutions to obtain a 1 M concentration (1 mol/L) of nitric acid at a pH = 0. Then, both solutions are mixed together. Using this method, all the oxide components of Bi and Te are completely dissolved in the electrolyte. Nitric acid is used because it can dissolve bismuth oxide and tellurium oxide so that H^+^ acts as a working ion and NO^3−^ acts as a counter ion. Meanwhile, SbTe thick films are grown by pulsed electrochemical deposition at room temperature and a stirring speed of 60 rpm. The electrolyte solution consists of 6 mM Sb^3+^, 3.6 mM HTeO^2+^, 0.5 M C_4_H_6_O_4_·5H_2_O, and 1 M HNO_3._ The oxide component of Sb is inert in the nitride acid. Therefore, tartaric acid is employed to dissolve the Sb_2_O_3_ and results in SbO^+^ and (C_4_H_4_O_6_)^2−^.

### 2.3. Characterization

Because a material property evaluation needs to be conducted on an insulating substrate to avoid short circuiting, the synthesized films are peeled from the substrate by epoxy resin and mounted on a glass substrate [17]. The in-plane Seebeck coefficient is measured at room temperature between two points of the film. The temperatures are observed by 50 µm diameter K-type thermocouples. Multiple measurements are carried out at many temperature differences from 4 to 8 °C to ensure accurate results. The Seebeck coefficient, obtained from the generated voltage as a result of given temperature gradient, is measured for these samples. The electrical resistivity is measured using a four terminal method. Synthesized films are imaged by Scanning Electron Microscopy (SEM) equipped with Energy Dispersive X-ray spectroscopy (EDX), which is used for composition analysis. These measurements are performed on a Field Emission Gun Scanning Electron Microscope Hitachi SU-70 (Hitachi, Tokyo, Japan). X-ray diffraction (XRD) patterns of the deposited films are recorded with an X-ray Diffractometer Bruker- D8 (Billerica, MA, USA) using Cu α radiation (λ = 1.5418 Å, 40 kV, 40 mA, step size 0.02°, 2 s/step, and with a sample rotation 60 rpm). The nanostructures of the sample are evaluated by High Resolution Transmission Electron Microscopy (HRTEM) and Selected Area Electron Diffraction (SAED) on a JEOL-2100F instrument (JEOL, Tokyo, Japan). The cross-sectional preparation is performed by a mechanical thinning and dimpling method, followed by Ar^+^ ion beam milling to make the area transparent to electrons.

## 3. Results and Discussion

### 3.1. Synthesis of N-Type Bismuth Telluride

#### 3.1.1. Voltammetry

The reaction equation for BiTe is [18]:

13H^+^ + 18e^−^ + 2BiO^+^ + 3HTeO_2_^+^ ↔ Bi_2_Te_3_ + 8H_2_O. (1)

**Figure 2:**
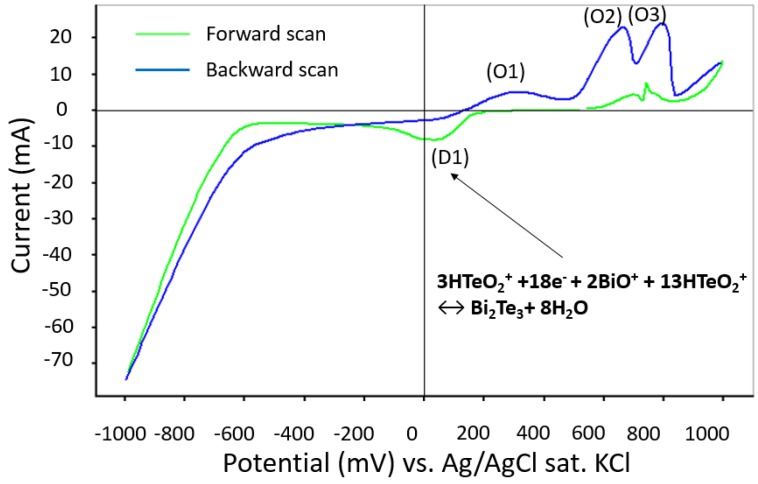
Cyclic voltammetry for BiTe deposition.

The cyclic voltammetry (CV) presented in Figure 2 is recorded between −1 V and 1 V with a scan speed of 10 mV/s. The literature states that, during the deposition process, the first main reduction relates to the formation of BiTe films [19,20]. In this work, the first reduction ranges between −0.1 V and 0.1 V. During the backward scan, three oxidation peaks, O1, O2, and O3, appear at 300 mV, 600 mV and 800 mV, respectively. Peak O3 belongs to the depriving process of Bi and Te on the gold surface. Peaks O1 and O2 represent an oxidation of the residual elemental Bi [19,20]. From the CV curve, the appropriate potentials for growing BiTe films are determined to be among the first reduction peak occurring at approximately 20 mV.

#### 3.1.2. Constant Deposition

**Figure 3:**
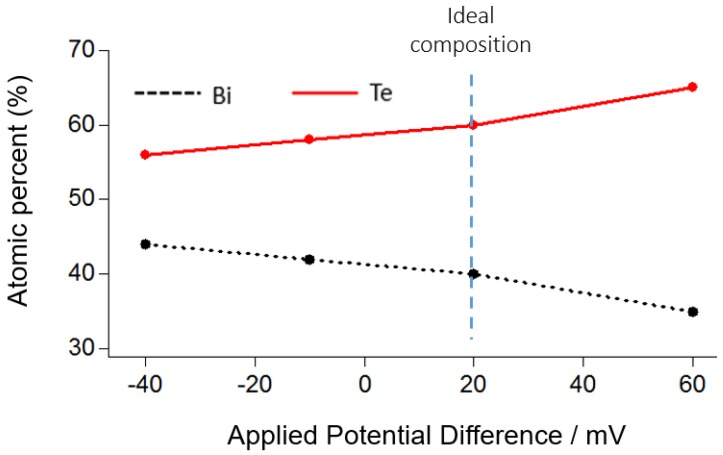
Potential dependence on the atomic composition of BiTe.

First, constant deposition is used to synthesize thick Bi_2_Te_3_ films. From the CV analysis, some deposition potentials in the first reduction range are examined to evaluate the potential dependence on the atomic composition, as shown in Figure 3. During the deposition process, BiTe stoichiometric formation is obtained from the balance of potential-dependent chemical kinetics for the deposition of both bismuth and tellurium [19]. That balanced stoichiometry is obtained at the potential where the first reduction peak of the electrolyte CV curve is observed, as shown in Figure 2. Because the reduction potential of tellurium is greater than that of bismuth, the atomic composition of tellurium is more advantageous than that of bismuth when the applied potential is larger than the balanced stoichiometry. In contrast, if the potential is smaller than that of balanced stoichiometry, more bismuth atoms will be deposited, as shown in Figure 3. From the results, 20 mV is determined to be the most balanced stoichiometry potential to electrochemically deposit Bi_2_Te_3_. At −40 mV, bismuth telluride is synthesized in the form of Bi_2.3_Te_2.7_, and Bi_1.8_Te_3.2_ appears at 60 mV.

**Figure 4:**
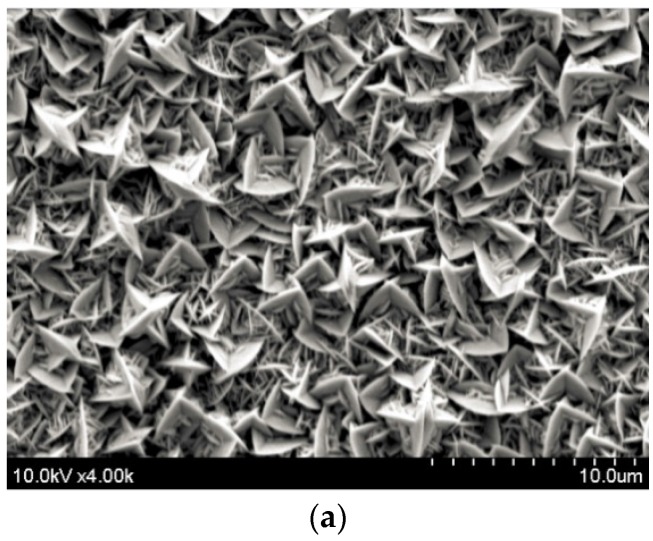

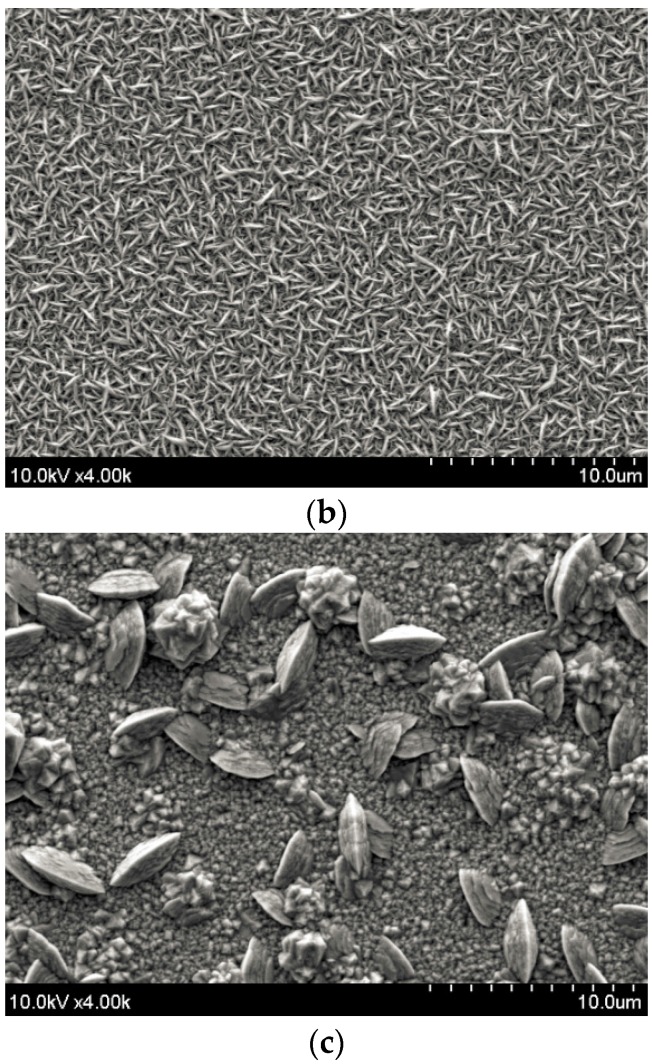
Surface morphology of the BiTe deposition potentials: (**a**) −40 mV; (**b**) 20 mV; and (**c**) 60 mV.

The deposition potential not only changes the atomic composition of the material but also has a significant effect on the crystal morphology. Figure 4 shows the deposition potential dependence of the surface morphology of the film. The sample synthesized with a potential of −40 mV exhibits a standing plate-like shape with large grains of approximately 4 µm. When the deposition potential increases to 20 mV, the grain size decreases (~1 µm) and changes to a granular structure for a deposition potential of 60 mV. An XRD measurement is also performed to analyze the effect of the deposition potential on the crystal growth orientation.

**Figure 5:**
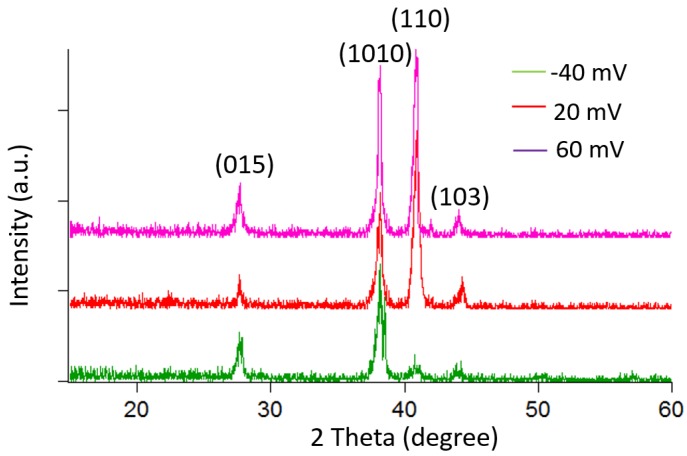
X-ray diffraction (XRD) pattern of the BiTe films for different deposited potentials.

Figure 5 shows the XRD patterns of the deposited bismuth telluride films, which exhibit a polycrystalline structure with (110), (1010) and (015) as the prominent diffracted peaks. However, the intensity ratios of the peaks are not similar for each deposition potential, indicating an effect on the growth orientation.

**Figure 6:**
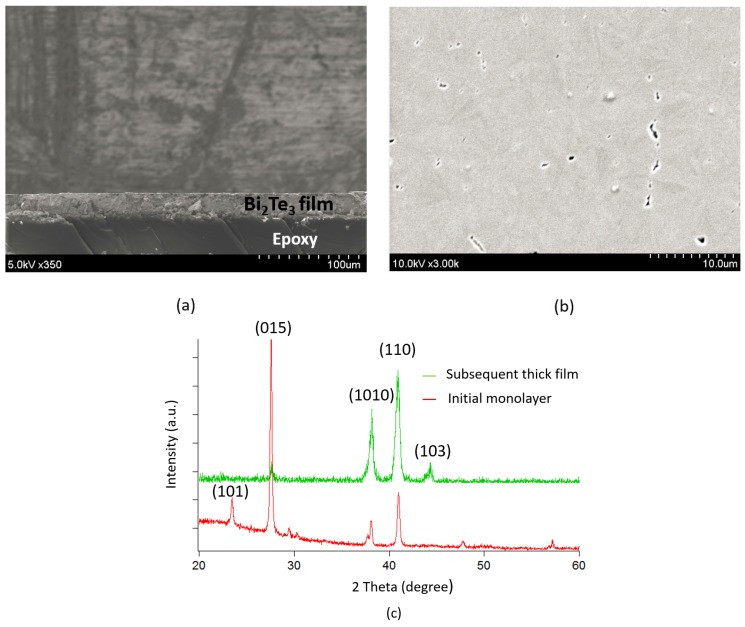
The dependence of the metallic seed layer on the lattice matching of the initial monolayer: (**a**) sample structure after being separated from the metallic seed layer with the monolayer on the top surface; (**b**) surface morphology determined via Scanning Electron Microscopy (SEM) observation; and (**c**) XRD pattern of the initial monolayer.

To research the dependence of the metallic seed layer on the lattice matching of the initial layer, the material film is separated from the Au- film interface using epoxy resin [17], as shown in Figure 6a. Subsequently, the sample is mounted on the glass wafer to perform measurements on the initial layer of the film. Figure 6b shows the SEM image of the interface obtained by above separation. Additionally, the XRD patterns illustrate the difference as the diffracted peak of (103) in the subsequent thick film is disappeared and a new peak of (101) is found in the initial matching layer, as shown in Figure 6c. It can be concluded that, although the growth orientation is slightly altered the crystal structure is unchanged.

**Figure 7:**
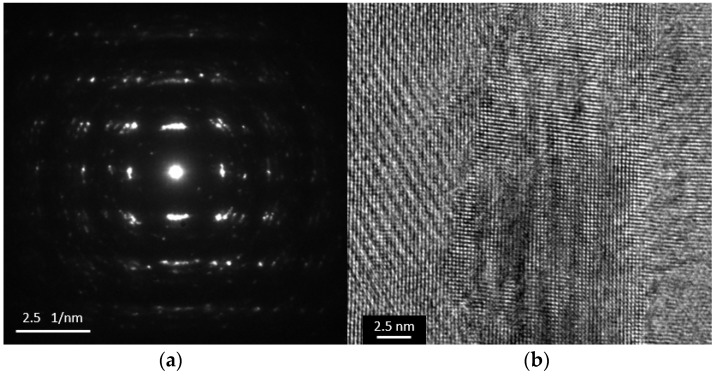
(**a**) Selected Area Electron Diffraction (SAED) pattern; and (**b**) High Resolution Transmission Electron Microscopy (HRTEM) observation between the boundaries of the Bi_2_Te_3_ sample.

**Figure 8:**
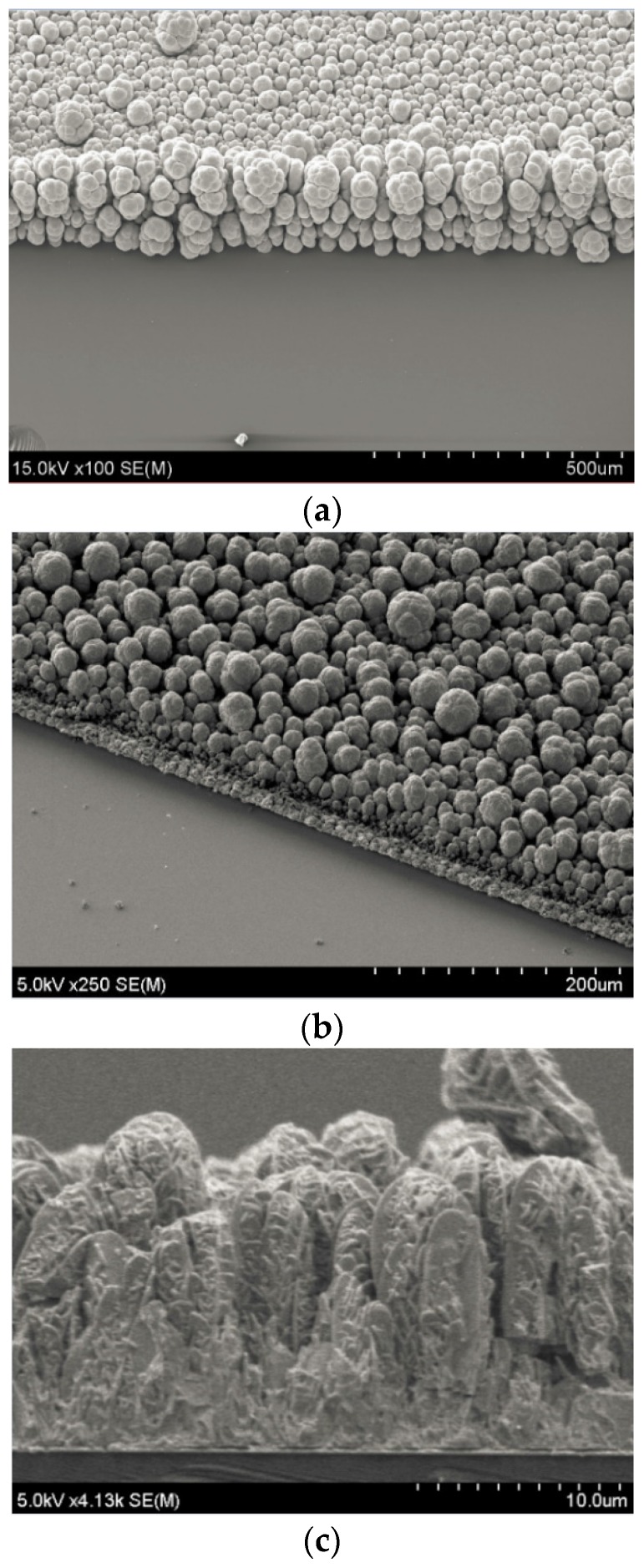
Morphology of the thick BiTe film synthesized by the constant potential method: (**a**) 200-µm-thick film; (**b**) cross-section view of the 200-µm-thick film consisting of two different layers; and (**c**) cross-section structure of the initial 10-µm-thick film.

The electron diffraction pattern and the HRTEM image are shown in Figure 7a,b, respectively. The SAED observation illustrates many diffraction patterns that overlap along the concentric circles, indicating grain texturing. It is concluded that the sample consists of polycrystalline nanograins. When the deposition is performed for a long time, the oxidation of Bi or the over-deposition of Te leads to a change in the stoichiometry and causes stressed or porous layers. These layers become unstable as the layer thickness increases. For this reason, only a small number of studies have been successful in synthesizing thick films of BiTe and SbTe [3,14,15,16,21]. As shown in Figure 8a, although a 200-µm-thick film can be grown using the constant method at a high deposition rate of approximately 30 µm/h, only 20 µm of the thickness is actually of good quality (Figure 8b). The thick film layer on the top contains porous structures or particles that easily peel from the underlying layer. The 10-µm-thick layer in Figure 8c exhibits an initial 4-µm-thick layer that has a compact structure. Upon further deposition, the porous structure begins to appear. To solve this problem, another method and technique must be considered when synthesizing a thick film.

#### 3.1.3. Pulsed Deposition

**Figure 9:**
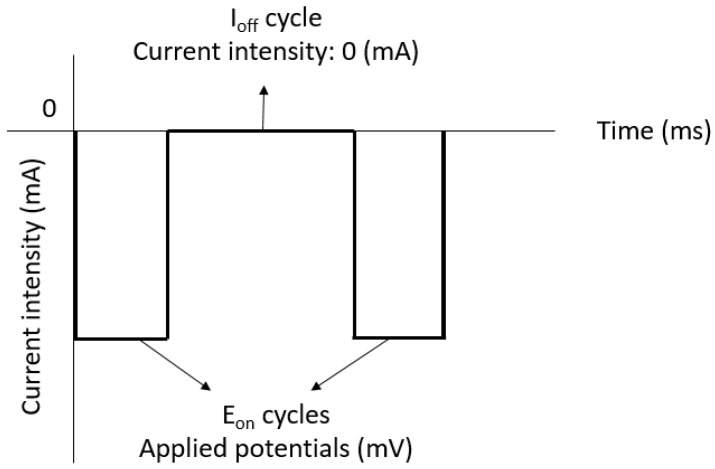
Pulsed deposition waveform.

To synthesize thick Bi_2_Te_3_ films, pulsed electrochemical deposition is performed using a waveform shown in Figure 9. The applied potentials used in the pulsed deposition alternate between E_on_ cycles and I_off_ cycles. At the E_on_ cycles, the potential condition of the potentiostatic mode is adjusted to grow Bi_2_Te_3_, as described in the constant deposition section. Meanwhile, the working electrode current is maintained at 0 mA during the I_off_ cycles.

**Figure 10:**
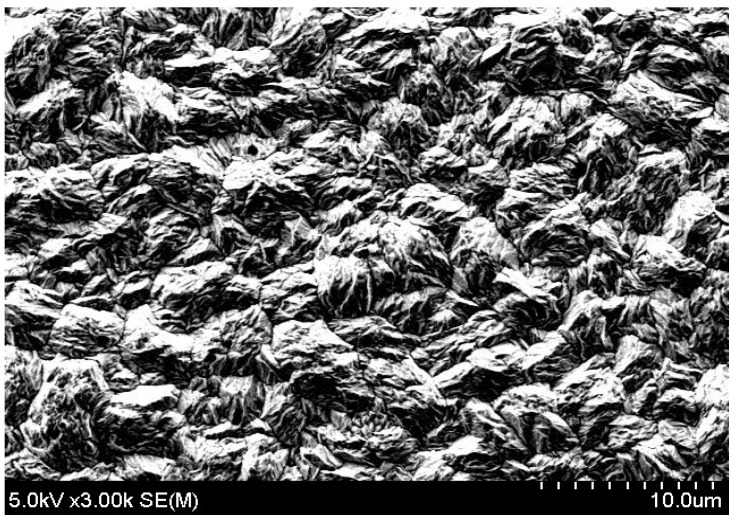
SEM image of Bi_2_Te_3_ film surface formed by pulsed deposition.

**Figure 11:**
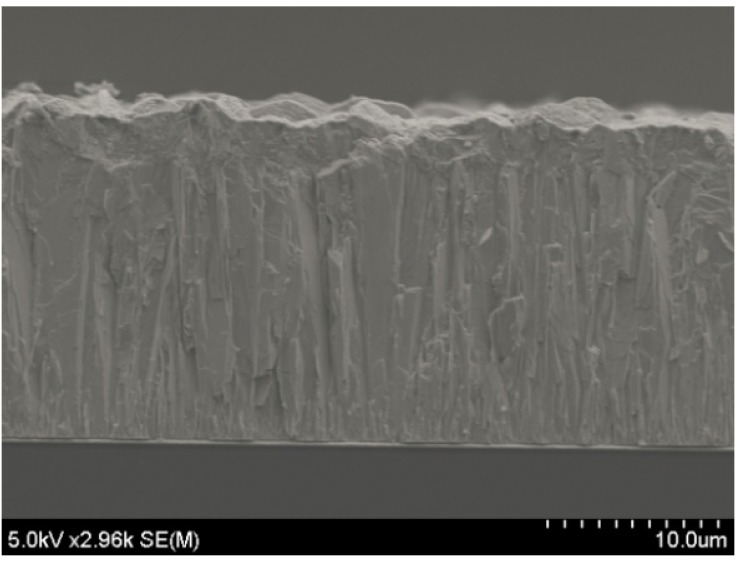
Cross-section structure of the film grown by pulsed deposition.

The working mechanism is as follows. During the deposition cycle with E_on_ = 20 mV, the reduction reaction can be precisely controlled to grow material and during the cycle with I_off_ = 0, any undesired reactions are prevented. The pulse width is set to 0.1 s for the ON status and 0.2 s for the OFF status. The advantages of the pulsed deposition over constant deposition is that it can balance the loss of ions at the surface of the working electrode that occurs when a constant potential is applied to ionize the electrolyte [22]. In principle, the pulsed deposition is used to ensure the amorphous material deposited during the ON period is in a position to crystallize during the OFF period. Significant improvements may be due to two different effects of the crystal growth: new crystals can grow on the surface as a homogeneous distribution, or atoms and ions can be added to any existing layers. A small surface diffusion and a high electrochemical potential possibly incite the growth of new crystals, whereas a high surface diffusion and a small electrochemical potential can promote the adhesion of formed atoms to existing layers. During pulsed electrochemical deposition, a better supply of material within the solution may result in crystal growth [23]. This is proven in Figure 4b and Figure 10. The surface of the Bi_2_Te_3_ deposited by the pulsed waveform is more uniform and smoother than that for a constant deposition. Although the surface of the sample prepared by pulsed deposition method is dense, it is rough and loose with dendritic growth for the constant deposition method. The 10-µm-thick sample synthesized by constant deposition shows a roughness of 2 µm. This is much larger than the 300-nm roughness of the sample formed by the pulsed deposition method. The crystal size of approximately 1 µm formed by constant deposition is apparently smaller than that of the pulse deposited crystals (~5 µm). This is related to the diffusion limitations during the constant deposition, since the lateral growth is restricted if enough ions are not supplied to the edges of the crystals. As a result, the new crystals will tend to grow on the top of the already formed crystals to create a standing shape, as shown in Figure 4b. Therefore, the size of the crystals becomes narrower. For the films deposited using the pulsed method, much wider crystal sizes are obtained, as shown in Figure 10. Again, the observation of the material cross-sections shown in Figure 11 confirms this explanation. The crystals deposited by pulsed deposition are formed uniformly to create a more reinforced compact structure than that from constant deposition. However, the effect of the pulsed deposition method will be limited if the deposition rate is too high during the ON period. A long OFF period is not really effective in this case. Therefore, a millisecond pulsed deposition is first presented in Reference [23] to solve this issue. The purpose of this method is to limit the high deposition rate during the ON period. In this work, we propose another approach of low concentration electrolytes. Additionally, the usage of soluble anodes can stabilize the deposition, but it may affect a low concentration of ions and cations. Therefore, electrolytes are renewed frequently to avoid a significant depletion of low concentrations of the species.

**Figure 12:**
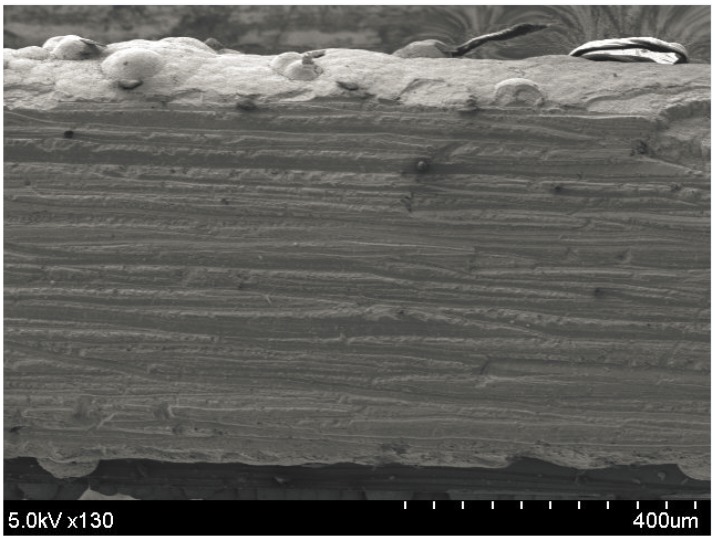
SEM image of the cross section of the 600-µm-thick Bi_2_Te_3_ film.

As a result, a film with a thickness of approximately 600 µm and single crystalline bulk-like structure is achieved, as shown in Figure 12. The deposition rate is approximately 10 µm/min. An XRD measurement is performed to analyze the crystal growth orientation of the synthesized films.

**Figure 13:**
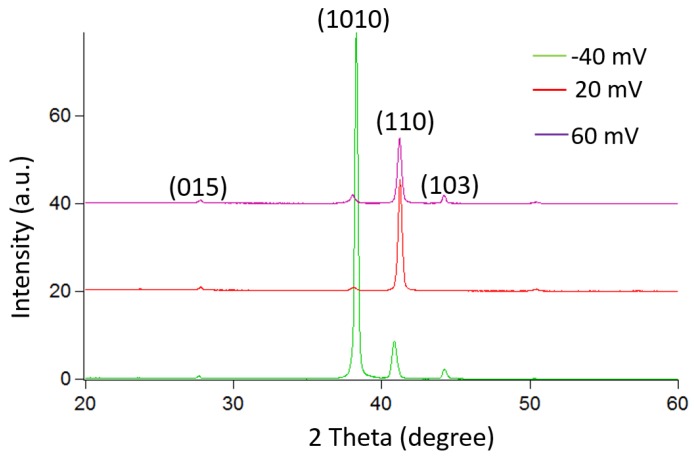
XRD pattern of the BiTe films with different deposited potentials.

The X-ray diffraction proves that the pulsed-deposited Bi_2_Te_3_ thick film possesses a Rhombohedral-hexagonal structure, as shown in Figure 13 [14]. The most intensive peak (110) occurs at 41° of two theta while peaks at (015) and (103) are eliminated and become almost unobservable. This result is in agreement with previous studies of Bi_2_Te_3_ thin films [19,24,25]. In comparison with constant deposited films, the diffracted peak intensity becomes much narrower. This change indicates a significant increase in the grain size and improved crystallinity. This again confirms the results from the SEM observation, which indicates the difference in crystal growth between the constant and pulsed deposition methods. It is clear that the Bi_2_Te_3_ films grown using constant deposition change from a polycrystalline to a (110) single crystal-like structure when using pulsed deposition.

**Figure 14:**
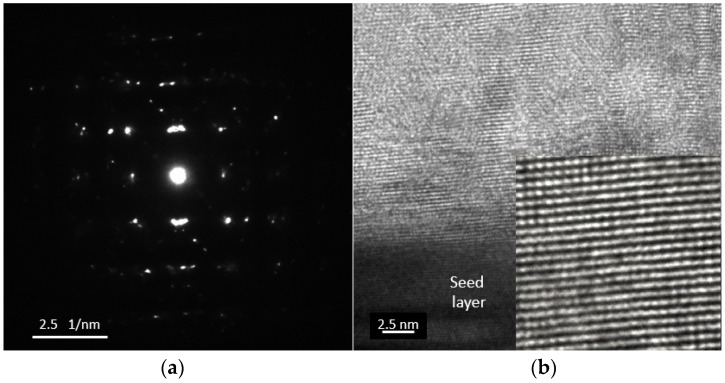
(**a**) SAED pattern; and (**b**) HRTEM observation between the boundaries of the Bi_2_Te_3_ sample fabricated using pulsed deposition.

In the conclusions from References [26,27], the figure of merit (ZT) is an anisotropic property. The single peak of the (110) highly oriented structure has the highest value of ZT. The (110) highly oriented structure yielded for thick films are one of the achievements from this research. In contrast to the constant deposition method, the clear SEAD pattern of the pulsed-deposited sample reveals that it basically possesses a single crystalline structure with a major Bi_2_Te_3_ phase, as shown in Figure 14a. From the HRTEM observation in Figure 14b, the distance between two neighboring faces is estimated to be approximately 2.2 Å, which is the same as the inter-planar distance between the (110) lattice planes.

#### 3.1.4. Thermo-Electric Properties Evaluation

The electrical resistivity (ρ) and Seebeck coefficient (*S*) are evaluated for the Bi_2_Te_3_ films deposited by both methods. The Seebeck coefficient of the sample deposited with the constant mode is observed to be a maximum of −60 µV/K. The pulsed-deposited Bi_2_Te_3_ film exhibits a slightly higher Seebeck coefficient with an average of −80 µV/K. However, the electrical resistivity of the films synthesized by pulsed deposition is much improved over specimens fabricated via constant deposition. The electrical resistivity of the samples prepared by the pulsed method exhibit a value of 20 µΩm. This is approximately 2.5 times lower than 50 µΩm from the constant method. The main reason for this difference is the aforementioned crystal growth structure. In a material containing defective grain boundaries, charge carriers may be scattered at the interfaces between grains. The cross-section structure observations, XRD and TEM analyses show that the sample deposited using the constant method contains more defects in the grain boundaries than for the pulsed deposition. This leads to a low electrical conductivity when using the constant deposition method. The relationship between Seebeck coefficients and electrical conductivities of BiTe and SbTe is a complicated issue. According to works reported, many behaviors of this relationship have been observed. In References [18,20], the relationship between Seebeck coefficients and electrical conductivities behaves differently when crystals growth is improved by pulsed deposition or annealing process. In detail, it is shown that Seebeck coefficients increase while electrical conductivities decrease in Reference [20]. In contrast, both properties are enhanced dramatically in Reference [18]. The same results are also reported that both Seebeck coefficients and electrical conductivities are much improved due to high quality crystallinity and low defect concentration [23,28].

**Figure 15:**
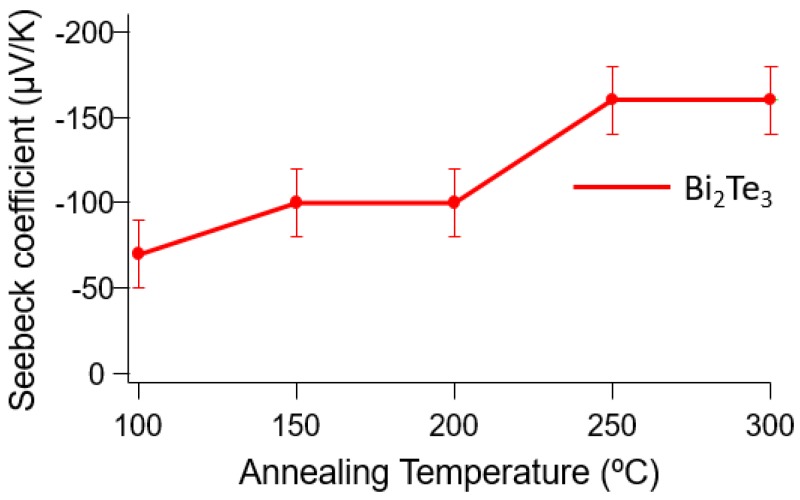
Dependence of the Seebeck coefficients on the annealing temperature.

Additionally, this paper reports how annealing affects the Seebeck coefficient and electrical resistivity of the Bi_2_Te_3_. The Bi_2_Te_3_ films are annealed at various temperatures in N_2_ at ambient conditions for 1 h. The heat ramp rate during the annealing process is 2 °C/min. Then, the films are analyzed using EDX to confirm that there is no elemental oxygen detected to prevent the formation of oxidation during the annealing process. The result shows that, although annealing can improve the thermal and electrical properties of materials, some annealing temperatures are found to optimize material performance. The highest Seebeck coefficient for the Bi_2_Te_3_ films is found at an annealing temperature of approximately 250 °C, as shown in Figure 15.

At a 250 °C annealing temperature, thermoelectric property measurements show that the annealing process can significantly improve Seebeck coefficient and electrical resistivity of the materials synthesized from both constant and pulsed deposition methods. The Seebeck coefficients of the films prepared using the constant and pulsed deposition methods are evaluated to be improved by a factor of roughly two after the annealing process. The annealed sample is measured to have a Seebeck coefficient of −110 µV/K, which is remarkably improved from the −50 µV/K in the as-deposited sample fabricated using constant deposition. For pulsed deposition, a similar increase is also observed when the Seebeck coefficient improves from −80 µV/K to −150 µV/K. The electrical resistivity of the sample synthesized using pulsed deposition is slightly improved from 20 µΩm to 15 µΩm. Meanwhile, the electrical resistivity of the annealed films produced using constant deposition is impressively reduced in comparison with the non-annealed films. This improvement may be attributed to decreases in the defects in the grain boundaries occurring from the annealing process. As explained in References [21,29], the rearrangement of crystal grains and removal of the defects are also the reasons for a similar improvement of the Seebeck coefficient due to the annealing process. The summarized results are shown in Table 1.

### 3.2. Synthesis of P-Type Antimony Telluride

#### 3.2.1. Voltammetry

**Figure 16:**
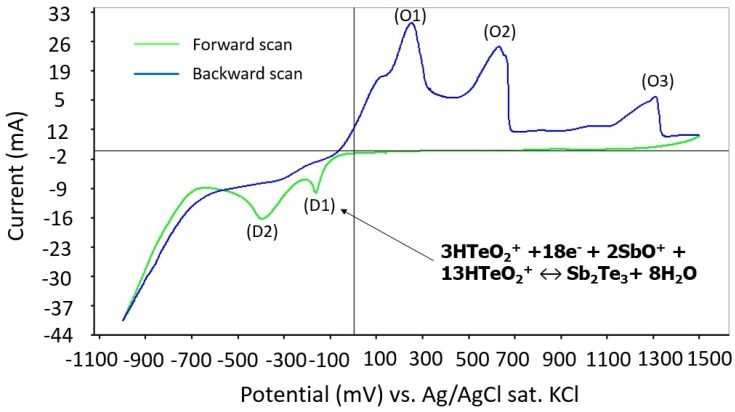
Cyclic voltammetry of SbTe deposition.

To understand the formation mechanism for SbTe during the deposition process, the cyclic voltammetry of the reactions is studied. The potentials are swept over a range from −1.0 to 1.5 V at a scan speed of 10 mV/s. According to the cyclic voltammetry results shown in Figure 16, two main reduction peaks (D1 and D2) and three oxidation peaks (O1, O2, and O3) are observed. The first main reduction peak, D1, is located at approximately −150 mV, which correlates to the formation of SbTe films, whereas the following reduction reaction appears at −450 mV because of hydrogen evolution [23,30]. The three oxidation peaks correspond to the process of stripping the Te atoms. Therefore, a potential range between −200 mV and 100 mV is determined to be an appropriate potential condition for depositing SbTe films on a gold film. The reaction taking place during the deposition process is shown to be [23]:

3HTeO_2_^+^ +18e^−^ + 2SbO^+^ + 13HTeO_2_^+^ ↔ Sb_2_Te_3_+ 8H_2_O. (2)

**Figure 17:**
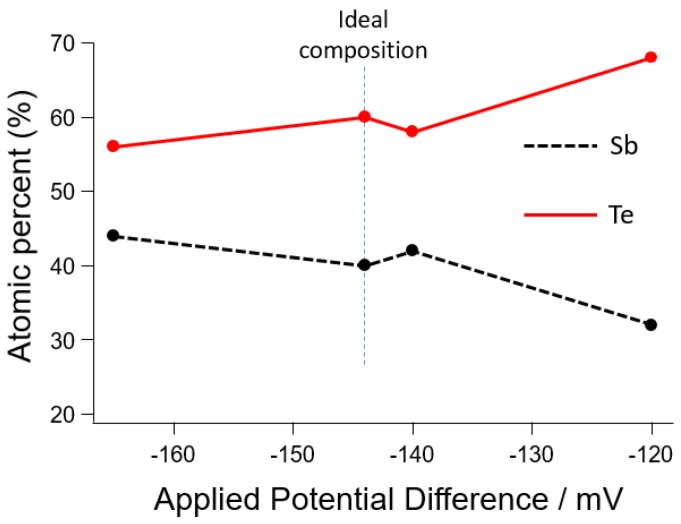
Potentials dependence on the atomic composition of the Sb_2_Te_3_.

The dependence between deposition potentials and atomic composition in the range of suitable potentials from the CV measurement is also investigated, as shown in Figure 17. The ideal composition of 40 atomic % Sb and 60 atomic % Te is achieved at a potential of -144 mV. In the SbTe formation, antimony is deposited at a higher potential than bismuth and in the region where the tellurium deposition potential is already large. Therefore, a stronger stoichiometry dependence on the deposition potentials than for BiTe is predicted.

#### 3.2.2. Pulsed Deposition

**Figure 18:**
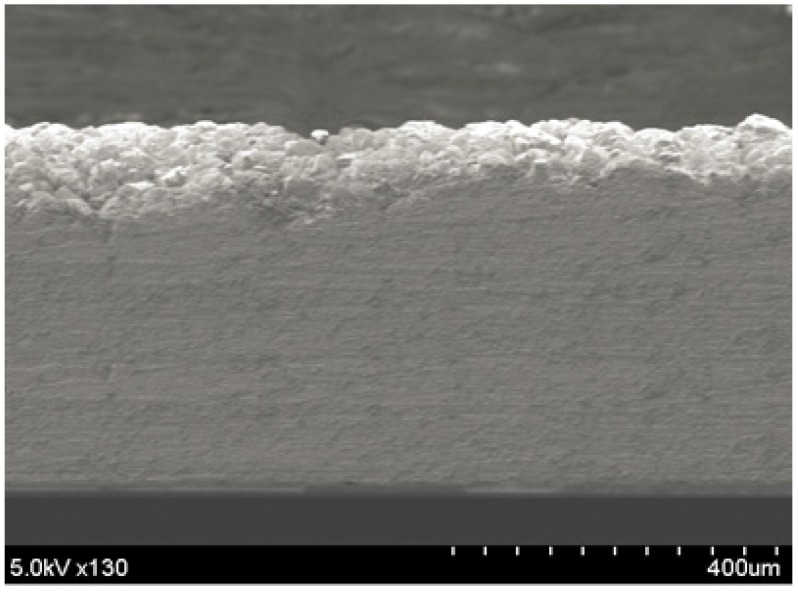
SEM image of the cross section of the 500-µm-thick Sb_2_Te_3_ film.

**Figure 19:**
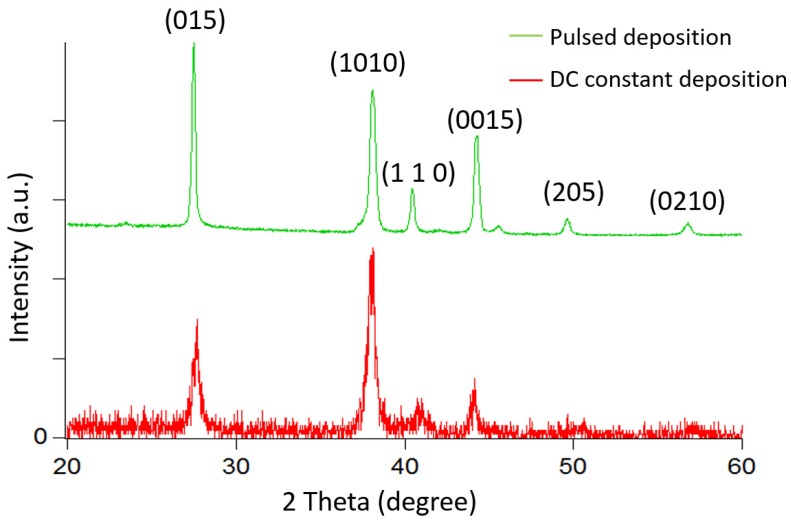
XRD patterns of the Sb_2_Te_3_ films synthesized by constant and pulsed deposition at −144 mV.

**Figure 20:**
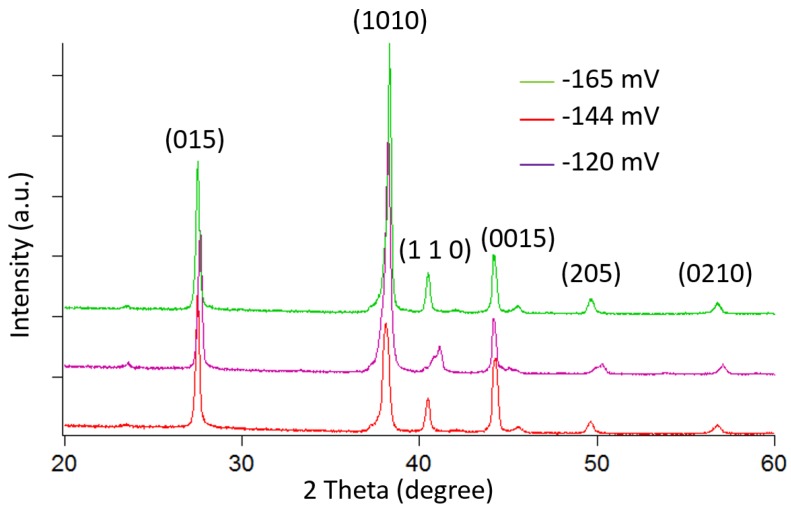
XRD pattern of the SbTe films at different deposited potentials.

Sb_2_Te_3_ thick films have been grown using pulsed electrochemical deposition at room temperature and a stirring speed of 60 rpm. The pulsed waveform is applied at −144 mV for the ON period for 0.1 s and a zero current during the OFF period for 0.2 s. After one hour, precipitation of Sb_2_O_3_ begins to appear. This phenomenon causes a loss of (C_4_H_4_O_6_)^2−^ ions because (C_4_H_4_O_6_)^2−^ is the result of the dissolution of Sb_2_O_3_ in tartaric acid. Therefore, the electrolyte is renewed for every hour of deposition. Finally, 500-µm-thick Sb_2_Te_3_ film is successfully synthesized with a deposition rate of approximately 4 µm/h, as shown in Figure 18. The crystal structure is analyzed using XRD for Sb_2_Te_3_ films deposited by both constant and pulsed depositions at −144 mV. The film synthesized by pulsed deposition exhibits narrower diffracted peak intensity than that of the constant method. However, both samples possess a polycrystalline structure, as shown in Figure 19. Similarly, all of the SbTe films synthesized at three different potentials near the first main reduction peak exhibit randomly oriented polycrystalline, as shown in Figure 20.

#### 3.2.3. Thermo-Electric Properties Evaluation

**Figure 21:**
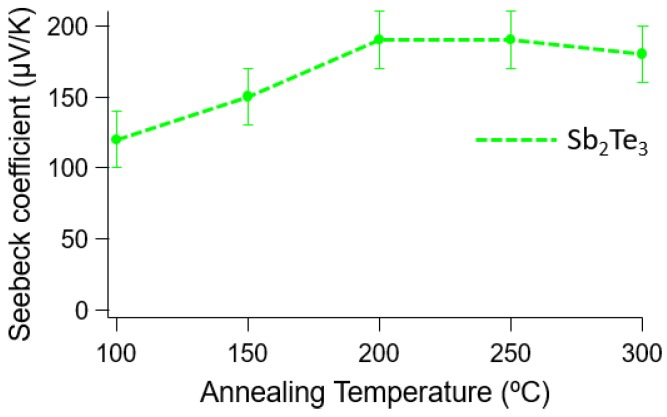
Dependence of the Seebeck coefficients from the Sb_2_Te_3_ on the annealing temperature.

The Sb_2_Te_3_ films are also annealed to improve the thermal and electrical properties. The annealing process is performed for 1 h in N_2_ atmosphere. The heat ramp rate of 2 °C/min is conducted during the annealing process. The effect of the annealing process on the Seebeck coefficient for Sb_2_Te_3_ is different from that of Bi_2_Te_3_. The Seebeck coefficient for the as-deposited Sb_2_Te_3_ sample exhibits a value of approximately 140 µV/K. After the annealing process, this increases to approximately 170 µV/K. However, the electrical resistivity is as much as three times better than that of the as-deposited films. The electrical resistivity remarkably decreases from 60 µΩm to 20 µΩm after the annealing process. The large value for the electrical resistivity possibly relates to the high number of defects at the grain boundaries. Therefore, the annealing process greatly affects the electrical resistivity. This is as same behavior as for the constant deposition of Bi_2_Te_3_ compared to the pulsed deposition. In this case, the film’s electrical resistivity in the sample produced by the constant method is more dramatically decreased by the annealing process than that for the pulsed deposition method because of its worse crystal defects. In comparison to the thin Sb_2_Te_3_ films reported on previously [23,31], the thick films synthesized in this work do not show a significant difference in the electrical resistivity than for the thin films. This proves that the thick films obtained in this work can have the same properties as thin films. The proper annealing temperature for the Sb_2_Te_3_ is found to be lower than for the Bi_2_Te_3_, which is approximately 200 °C, as shown in Figure 21. Summarized results are shown in Table 2.

## 4. Conclusions

In this report, the syntheses of thick bulk-like thermoelectric Bi_2_Te_3_ and Sb_2_Te_3_ materials are demonstrated for such applications as micro thermoelectric power generators. N-type Bi_2_Te_3_ and P-type Sb_2_Te_3_ thick films are obtained. A new usage of low concentration electrolyte is proposed as an approach for the synthesis of materials with high quality morphology and compactness. The conditions and effects of the annealing process are also investigated. Both materials exhibit a high Seebeck coefficient and low electrical resistivity. The Seebeck coefficient of the synthesized thermoelectric materials can reach approximately ±150 µV/K. Electrical resistivities of 15 ± 5 µΩm and 25 ± 5 µΩm are obtained for 600-µm-thick Bi_2_Te_3_ and 500-µm-thick Sb_2_Te_3_ films, respectively.

## Figures and Tables

**Table 1:** Effects of annealing on the Bi_2_Te_3_ properties.

	Constant Deposited Bi_2_Te_3_		Pulsed-Deposited Bi_2_Te_3_	
	Non-Annealing	Annealing (250 °C)	Non-Annealing	Annealing (250 °C)
Seebeck coefficient (±20 µV/K)	−50	−110	−80	−150
Electrical resistivity (±5 µΩm)	50	20	20	15
Power Factor (W/mK^2^)	0.5 × 10^−4^	6 × 10^−4^	3.2 × 10^−4^	15 × 10^−4^

**Table 2:** The effects of annealing on the Sb_2_Te_3_ properties.

	Pulsed-Deposited Sb_2_Te_3_	
	Non-Annealing	Annealing (200 °C)
Seebeck coefficient (±20 µV/K)	130	170
Electrical resistivity (±5 µΩm)	60	25
Power Factor (W/mK^2)^	2.8 × 10^−4^	11.2 × 10^−4^
